# Cost-Effective Bioimpedance Spectroscopy System for Monitoring Syncytialization In Vitro: Experimental and Numerical Validation of BeWo Cell Fusion

**DOI:** 10.3390/mi15121506

**Published:** 2024-12-18

**Authors:** Karim Saadé, Mohammed Areeb Hussain, Shannon A. Bainbridge, Raphael St-Gelais, Fabio Variola, Marianne Fenech

**Affiliations:** 1 Department of Mechanical Engineering, University of Ottawa, Ottawa, ON K1N 6N5, Canada; ksaad019@uottawa.ca (K.S.); mhuss063@uottawa.ca (M.A.H.); raphael.stgelais@uottawa.ca (R.S.-G.); 2 Faculty of Health Sciences, University of Ottawa, Ottawa, ON K1S 5S9, Canada; shannon.bainbridge@uottawa.ca; 3 Department of Physics, University of Ottawa, Ottawa, ON K1N 6N5, Canada

**Keywords:** bioimpedance spectroscopy, COMSOL (Version 6.2) multiphysics, electrical capacitance, syncytiotrophoblast, transepithelial/transendothelial electrical resistance (TEER)

## Abstract

The placenta plays a critical role in nutrient and oxygen exchange during pregnancy, yet the effects of medicinal drugs on this selective barrier remain poorly understood. To overcome this, this study presents a cost-effective bioimpedance spectroscopy (BIS) system to assess tight junction integrity and monolayer formation in BeWo b30 cells, a widely used model of the multinucleated maternal–fetal exchange surface of the placental barrier. Cells were cultured on collagen-coated porous membranes and treated with forskolin to induce controlled syncytialization. Electrical impedance was measured using an entry level impedance analyzer, while immunofluorescence staining was used to confirm monolayer formation and syncytialization. The measurements and staining confirmed the formation of a confluent monolayer on day 4. In fact, the electrical resistance tripled for treated samples indicating a more electrically restrictive barrier. This resistance remained constant for treated samples reflecting the intact barrier’s integrity over the next 3 days. The measurements show that, on day 4, the electrical capacitance of the cells decreased for the treated samples as opposed to the untreated samples. This reflects that the surface area of the BeWo b30 cells decreased when the samples were treated with forskolin. Finally, a COMSOL model was developed to explore the effects of electrode positioning, depth, and distance on TEER measurements, explaining discrepancies in the literature. In fact, there was a substantial 97% and 39.4% difference in the obtained TEER values. This study demonstrates the AD2 device’s feasibility for monitoring placental barrier integrity and emphasizes the need for standardized setups for comparable results. The system can hence be used to analyze drug effects and nutrient transfer across the placental barrier.

## 1. Introduction

During pregnancy, the placenta facilitates the exchange of endogenous and exogenous substances, including nutrients and oxygen, between the maternal and fetal circulatory systems [1,2]. This temporary, yet crucial, organ plays a fundamental role in fetal development and the maintenance of pregnancy [3]. The fully developed human placenta is characterized by two functional compartments: the extravillous compartment, where a population of extravillous cytotrophoblast cells (EVTs) anchor the placenta to the uterine wall and ensure sufficient utero–placental blood flow; and, the villous compartment which serves as the site of maternal–fetal exchange and selective barrier function [4,5]. Highly branched, tree-like structures, the chorionic villi, are the functional exchange units within the villous compartment. The stromal core of these structures surrounds the fetal vasculature, encased by an incomplete layer of villous cytotrophoblast cells (CTBs) which, upon terminal differentiation, fuse into the overlying multinucleated syncytiotrophoblast (STB) cellular layer—a process known as syncytialization [6]. Maternal blood is brought into the surrounding intervillous space, where it bathes the STB of the chorionic villi, permitting the maternal and fetal circulations to come in proximity. Across this selective barrier, oxygen, nutrients (e.g., amino acids, vitamins, and glucose) and maternal antibodies are transported into the fetal circulation, while simultaneously preventing the passage of most pathogens and xenobiotics, safeguarding the developing fetus. As such, the structural and functional maintenance of this barrier, and particularly that of the multinucleated STB layer in direct contact with maternal blood, is essential for fetal development and a healthy pregnancy.

In certain situations, the use of pharmaceuticals during pregnancy becomes necessary, particularly in patients with conditions such as diabetes, epilepsy, or bipolar disorders [7]. However, our knowledge of transplacental drug transport and the potential risks to fetal development are limited by the obvious ethical constraints of clinical trials in human pregnant populations [8] and the clearly described differences in the placental structure and pharmacokinetics between humans and the animal species that are commonly used in pre-clinical investigations [9,10,11]. This limitation has driven the exploration and development of novel in vitro models to study human placenta development and function, including those with high-throughput capabilities tailored to pharmacological and toxicological studies [12,13].

Various trophoblast cell lines and in vitro models have been developed to study the maternal–fetal interface, with 2D models being the most widely used. These models are valuable for investigating placental hormone secretion, nutrient transport, and cytotoxicity due to their simplicity and established protocols [3,14,15]. However, their inability to replicate the structural complexity of the placenta has driven the development of 3D models incorporating trophoblast cell lineages and extracellular matrices (ECM) [16,17]. For instance, Kreuder et al. bioprinted a semi-permeable GelMA membrane seeded with primary placental fibroblasts to mimic chorionic villous stroma. By culturing BeWo cells on one side and vascular endothelial cells on the other, they assessed placental barrier permeability [18]. The choice of cell is crucial in placental models. While primary trophoblast cells are the gold standard, their limited availability, variability, and differentiation tendencies restrict their use in high-throughput assays [19]. Consequently, immortalized cell lines like HTR-8/SVneo [20], JAR [21], JEG-3 [22] and BeWo [23] are preferred. Among these, the BeWo cell line is considered to be the most suitable for modeling the maternal-facing placental barrier due to its ability to replicate cytotrophoblast terminal differentiation, fusion into multinucleated STB, and transplacental transport processes [3]. Hence, the well-established BeWo cell line was adopted in the current proof-of-concept study.

To visualize the tight junctions and the syncytialization process of the cells to determine the creation of a tight barrier, the cells need to be fixed, permeabilized, and stained. Therefore, the use of non-invasive techniques allows for the continuous monitoring of the barrier integrity without damaging the cells and the barrier [24]. One technique allowing such monitoring is called transepithelial/transendothelial electrical resistance (TEER). TEER measures the electrical resistance across a cellular monolayer and is a technique to non-invasively monitor barrier integrity and syncytialization during the various stages of cell growth and differentiation [25]. By correlating the electrical properties of the epithelial layer, TEER allows the measurement of cellular thickness, tight junction formation, cell layer confluency, and morphology [26]. However, biological tissues are able to impede electrical current, allowing the measurement of the electrical property of the tissue. In fact, bioimpedance spectroscopy (BIS) provides a more accurate representation of the electrical properties that biological cells exhibit. It not only allows the measurement of TEER but also of the electrical capacitance of the cell. The response of the electrical excitation, via current or potential, of the biological tissue can be measured using electrodes. By applying an alternating current, or AC, with a frequency sweep, the electrical impedance (Z) can be calculated as the ratio of the voltage (V) to the current (I). The electrical impedance is composed of resistive (R), capacitive (C), and inductive (L) components of the system and biological tissue [27]. Two resistive pathways are generated in biological tissues. The physical properties of biological tissues, composed of cells with membranes, surrounded by extracellular fluids, create two electrically conducting compartments: the paracellular (or extracellular) and transcellular (or intracellular) media, as described in Benson et al. [28,29,30].

Given the complex geometry and the variations associated with biological systems, TEER values can vary significantly, even for the same type of cells and similar growth conditions [3,19]. The cause of this variation has not been fully reported in the literature. Therefore, the use of simulations helps streamline studies and provides more objective comparisons and deeper insights, which cannot be fully understood experimentally. For instance, assessing the current flow characteristics in TEER measurements, the effect of environmental and experimental factors, or a combination of both is not possible experimentally. Khire et al. studied the effect of custom silicon nanomembranes to analyze TEER values, while Profka et al. studied the sensitivity of TEER values in different regions of the transwell setup [31,32]. This presents a need for a strong, experimentally validated simulation to better understand the experiments being conducted. In order to better understand TEER measurements and the flow of current throughout the system, FEM models have been developed. Yeste et al. studied the effect of geometry on sensitivity [33]. Khire et al. studied the effect of silicon membrane geometry on the sensitivity of TEER measurements and discussed the geometric correction factor (GCF) [31].

Therefore, to overcome the above downfall, we present a cost-effective BIS system to monitor tight junction and monolayer formation. This is accomplished through an experimental approach and validated using numerical methods. Briefly, a monolayer of BeWo cells was formed on a porous membrane coated with collagen. The BeWo cells were promoted to undergo syncytialization via forskolin treatment [34]. Electrodes and a 3D, custom-made support were used to measure the electrical impedance using the Analog Discovery 2 (AD2) device. A COMSOL model was used to replicate the experimental setup for the validation and accuracy analyses. Moreover, the COMSOL model was also used to explain the wide range of TEER values reported across the literature, by understanding the effect of various parameters on TEER values. This cost-effective system can further be utilized to analyze the effects of xenobiotics (e.g., pharmaceuticals) on placenta barrier integrity, as well as the transport of these chemicals and/or nutrients across the placental barrier. This system also demonstrates a potential to be applied to other culture models representing different epithelial barriers that rely on tight junction formation (i.e., intestinal epithelium); finally, it can be used in microfluidics channels for a more physiological and controlled environment.

## 2. Materials and Methods

### 2.1. Cell Culture

The human cytotrophoblast (BeWo b30) cell line was provided by Dr. Alan Schwartz (Washington University in St. Louis, MO, USA) and routinely cultured in 75-cm^2^ flasks at 37 °C and 5% CO_2_ using a DMEM/F-12 medium supplemented with 1% Penicillin–Streptomycin (P/S) (ThermoFisher, Waltham, MA, USA), 10% Fetal Bovine Serum (FBS) (ThermoFisher), and 1% Glutamax (ThermoFisher, Waltham, MA, USA). The cells were subcultured when they reached ~80% confluency in the flask and the media were replenished every other day.

### 2.2. Monolayer Formation on Porous Membrane

Polyester transwell inserts (pore size 0.4 um, growth area 1.12 cm^2^, apical volume 0.5 mL, basolateral volume 1.5 mL; cellQART—Sterlitech, Auburn, WA, USA) were pre-coated with 0.1 mg/mL mouse collagen Type IV for 2 h at 37 °C and 5% CO_2_ (200 uL on the apical side). Then, the membrane was washed 3× times with phosphate buffer saline (PBS). Cells were seeded at a density of 100,000 cells/cm^2^ and allowed to form a monolayer. The inserts were cultured for 7 days at 37 °C and 5% CO_2_ and the media were changed daily.

### 2.3. BioImpedance Spectroscopy (BIS)

**Figure 1:**
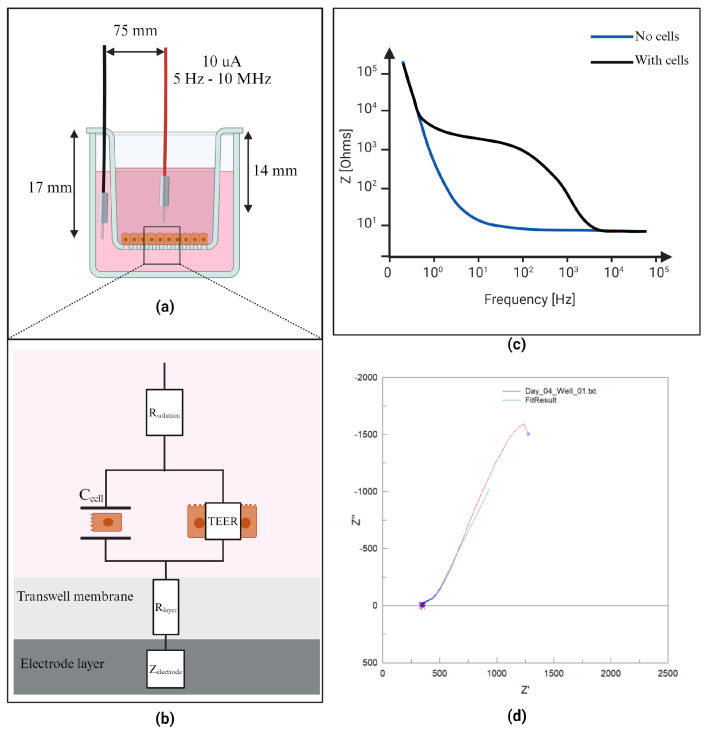
Experimental design: (**a**) electrode setup, (**b**) electrical model, (**c**) theoretical fitting of raw data. (**d**) experimental fitting of raw data based on electrical model.

Tight junction presence was assessed using BIS measurements under open circuit using a chopsticks electrode (STX4, World Precision Instruments, Sarasota, FL, USA). To ensure the stability of the reading and the reproducibility of the measurements, a 3D-printed support was utilized to secure the electrode and prevent any unintended movement that could potentially interfere with the measurements or cause damage to the cell layer. The first electrode tip was positioned at the center of the apical chamber, while the second electrode was placed in the basolateral chamber, approximately 7.5 cm away from the first electrode. The height of the electrodes, measured from the support, extended approximately 14 mm into the apical chamber and 17 mm into the basolateral chamber. The configuration of the electrodes and the experimental setup can be seen in Figure 1a. The Analog Discovery 2 (AD2) (Digilent, Pullman, WA, USA) generated a constant AC signal at 10 mA with a frequency sweep between 5 Hz and 1 MHz. Using ZView software (Version 4.0f) and the equivalent electrical model shown in Figure 1b,c, the TEER and the cell capacitance were extracted by fitting the raw data. The BIS was determined on the collagen-coated inserts in the presence or absence of cells over a 7-day period. The TEER and capacitance values for the cell layer were obtained by subtracting the intrinsic resistance (blank insert membrane) from the total resistance (insert membrane with cells) and were corrected for the surface area (Ω cm^2^).

### 2.4. Immunofluorescence Staining

After the completion of the cell culture experiments, the media were removed from the wells and washed 3 times using PBS. The cells were fixed by introducing 4% paraformaldehyde (PFA, Thermo Scientific, Waltham, MA, USA) into the transwells and incubated for 20 min at room temperature (RT), followed by 3 washes with PBS for all the immunofluorescence experiments. The transwell inserts were cut from the well and cut into 4 pieces. The cells were then permeabilized using 0.2% Triton-X (Sigma, San Diego, CA, USA) and incubated at room temperature for 20 min. After blocking with 5% donkey serum overnight at 4 °C, the cells were incubated with the primary antibody against E-cadherin (ThermoFisher Scientific, Waltham, MA, USA) and diluted at 1:500 in 2% donkey serum and PBS solution at 4 °C overnight. Subsequently, the cells were washed 3 times with PBS and treated with the appropriate secondary antibody (Alexa 647) (ThermoFisher Scientific, Waltham, MA, USA), diluted at 1:500 in PBS containing 2% donkey serum for 1 h at room temperature. The cells were also stained with DAPI at 1:1000 and then washed with PBS 3 times. The samples were finally mounted on #1.5 coverslips using VectaShield. Fluorescence imaging was performed using an AxioObserver Z1 inverted microscope (Zeiss, Oberkochen, Germany).

### 2.5. BeWo b30 Cell Fusion Assessment

Forskolin (Cederlane) was used to induce BeWo b30 differentiation to the syncytiotrophoblast. The media were supplemented with 20 μM of forskolin on day 2 post-seeding. The image collections were deconvoluted with ZenPro Software (Zeiss, Oberkochen, Germany, Version 3.0) and focus stacked with ImageJ (Version 2.14.0) (NIH, Bethesda, MD, USA). The files were then run through a CellProfiler (Version 4.2.1) pipeline to quantify the number of nuclei. The remaining cells were then manually counted using QuPath (Version 0.4.3) to assess the cell fusion index using Equation (1).

(1) $$FUI=\frac{\sum n_{f}}{N}$$

where $n_{f}$ is the number of multinucleated cells in a field, and *N* is the total number of nuclei of the same field.

### 2.6. FEA Model

A computational model of the transwell (cellQART—Sterlitech, Auburn, WA, USA) insert was simulated in COMSOL Multiphysics (Version 6.2) using the Electric Currents Module, with a mesh of ~8 × 10^5^ tetrahedral elements. A DC current of 10 µA was passed through the electrode to study the electrical resistance of the system [31]. Custom materials were created in the software, with the properties seen in Table 1. The conductivity of the semi-permeable membrane was calculated using the formula suggested by Yeste et al. [33]. The electrode was set as silver from the available material library in COMSOL. Additionally, the transwell insert was set as an insulator. After the validation of the COMSOL model using the TEER values obtained from the experimental approach, additional parameters, such as the distance between electrodes and the change in the height of the electrode in the basolateral well were studied to assess the impact of these factors on the TEER values.

### 2.7. Statistical Analysis

The number of samples for each experiment was determined on the basis of a minimum of three independent plates with three wells. The data were presented as the mean ± SD. A paired *t*-test was performed to determine if there were significant differences among the two different conditions: forskolin-treated and untreated samples.

## 3. Results and Discussion

### 3.1. Monolayer Formation and Assessment

**Figure 2:**
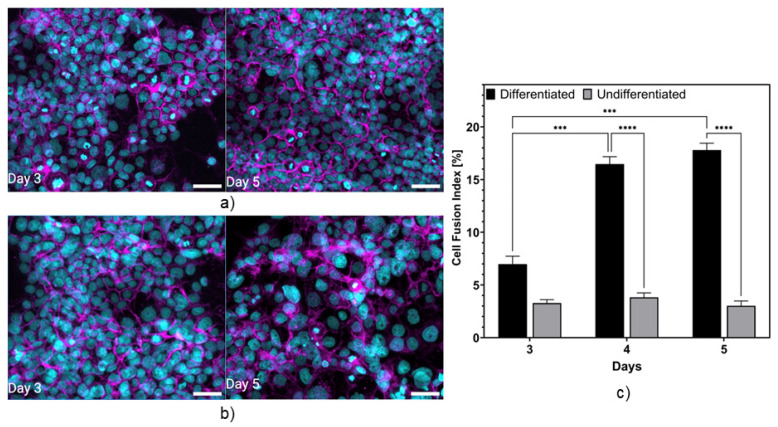
Representative fluorescent images of untreated (**a**) and forskolin-treated (**b**) BeWo cells after 3 and 5 days of culture. Scale bar: 55 μm and (**c**) quantification of cell fusion index. *** and **** indicate *p* ≤ 0.001 and *p* ≤ 0.0001, respectively.

Figure 2a,b show the immunofluorescence images acquired on days 3 and 5 post-seeding of the BeWo cells cultured under normal conditions (control: undifferentiated, pre-syncytialization) and following treatment with forskolin (differentiated, post-syncytialization). For the control samples, a monolayer was observed on day 5, with the cells qualitatively showing a strong expression of E-cadherin, resulting from the formation of a confluent layer and continuous intercellular tight junctions. When treated with forskolin (Figure 2b), the expression of E-cadherin was weaker, representing a significant loss of intercellular tight junctions, particularly by day 5. In addition, the cell nuclei observed were bigger, suggesting cell fusion, as confirmed in Blundell et al. [1]. Figure 2c shows the fusion index for the treated and untreated cells. The cell fusion, calculated from Equation (1), for the untreated wells on days 3, 4, and 5 showed no increase. On the other hand, there was an increasing trend observed over days 3, 4, and 5 for the treated cells from 7.6% to 17.3%. These results confirm that cell fusion occurs at a higher rate when cells are treated with forskolin.

Notably, the cell fusion index obtained was lower than that reported in the literature, likely because of the lower concentration of forskolin utilized in this study. In fact, Matsukawa et al. demonstrated a BeWo cell fusion index of 15%, in line with the current study, when promoting differentiation with 50 μM [35], whereas Azar et al. demonstrated a cell fusion index that surpassed 36% when 100 μM of forskolin was used [36]. No studies to date have demonstrated a 100% fusion rate, consistent with numerous reports that not all BeWo cells will fuse in culture, and it should be noted that there are noted differences in the methods used by the different groups in the calculation of the fusion index. Wang et al. further noted that the discrepancies in the reported fusion index may result due to the following: (1) differences in the types of CTB cells being studied, with isolated primary CTBs demonstrating a higher fusion rate compared to the transformed BeWo cell line; and (2) the immunofluorescent protocols being used, with discrepancies in the ability to adequately visualize the E-cadherin-stained boundary [37]. Moreover, other factors contributing to a higher fusion index could be attributed to media composition, the method used for the cell isolation, and, if using a primary cell line, the individual differences derived from the placenta donor [37]. It is important to note as well that the use of forskolin reduces cell proliferation, which could pose challenges to other cellular properties. In fact, Al-Nasiry et al. demonstrated that forskolin treatment led to a reduced proliferation rate and cell viability in BeWo cells. Their findings revealed a significant decrease in cell proliferation, with rates decreasing from 4% in control cells to less than 0.7% in forskolin-treated cells [38].

In conclusion, the results obtained from the cell fusion index analysis have shown the ability of BeWo to terminally differentiate and syncytialize in culture. This is significant as cell differentiation from CTBs to STBs is a key feature of the human placental barrier, with the formation of a structurally sound multinucleated STB layer responsible for regulating maternal–fetal exchange and the transplacental transfer of xenobiotics [2]. This section of the report validates the integrity of the barrier, which will be reflected in the impedance spectroscopy measurements.

### 3.2. Electrical Properties of BeWo b30 Monolayer

**Figure 3:**
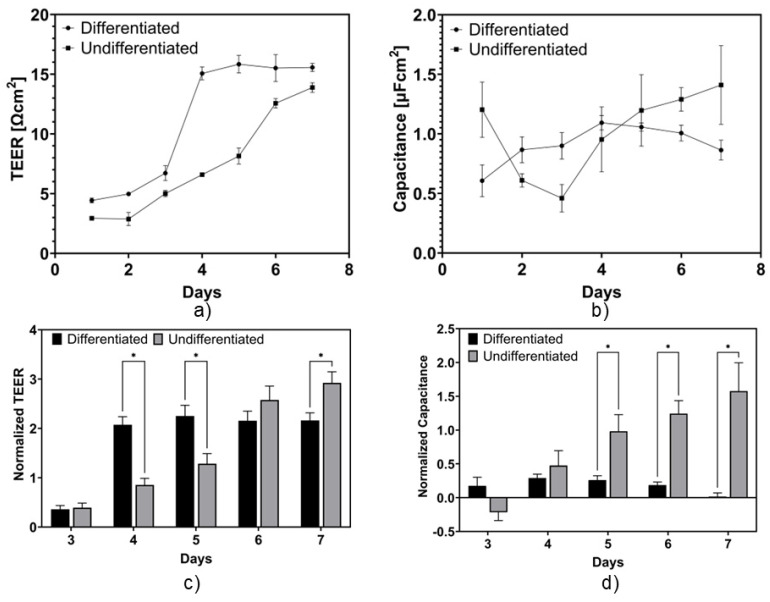
Impedance spectroscopy measurements and respective statistical analyses: (**a**) TEER, (**b**) electrical capacitance, (**c**) TEER rate of change with respect to day 2, and (**d**) electrical capacitance rate of change with respect to day 2. * indicates *p* ≤ 0.05.

Figure 3a shows the development of the TEER by BeWo for collagen Type I-coated transwell substrates. As can be seen, the control group showed a progressive increase in response over the course of the experiment. On the other hand, the samples treated with forskolin showed a different trend. The TEER remained low before reaching a peak on day 4, indicating the formation of a confluent monolayer, as previously confirmed with immunofluorescence imaging. The TEER value for the treated samples on day 4 was 3 times higher than the untreated samples, representing a more electrical-current-restricted, multinucleated structure.

As previously mentioned, forskolin treatment reduces cell proliferation. This is reflected in the measurements, in which the TEER values remained constant after the formation of a confluent monolayer, as opposed to the linearly increasing trend of the untreated samples. In addition, given that the cells were attached to the substrate, reflected by the constant TEER values on days 5, 6, and 7, the BeWo monolayer retained its integrity. On the other hand, the resistance of the untreated samples remained in an increasing linear trend due to their lack of fusion and multiple layer formation. This can be explained by referring to the immunofluorescence imaging. In fact, the untreated cells expressed more E-cadherin protein than the treated cells, indicative of the presence of intercellular tight junctions.

Moreover, due to their epithelial nature, BeWo cells start to grow in multiple layers. Therefore, the TEER of the untreated cells continuously increased over the days without any cell fusion changes, as shown in the previous section. Although the TEER values on day 6 for both the treated and the untreated cells were similar, the morphologies of the cells were different, as was shown in the immunofluorescence images. It was shown that there was a statistically significant difference between days 4, 5, and 7, confirming the effect of forskolin on the TEER values, as shown in Figure 3c. While the TEER values observed in this system may appear relatively low when compared to the findings from other studies, it is crucial to acknowledge that variations in instrumentation, temperature, buffers, growth substrates, and other lab-specific factors could contribute to these discrepancies. However, the trend of the measured values is similar to the other findings reported in the literature, indicating that the measurement device developed in this study is suitable to investigate BeWo barrier integrity for future studies, including more complex investigations of cell–cell interactions using co-culture models, or high-throughput pharmacokinetic studies examining the effects of various pharmaceuticals on the integrity of the placental barrier [19,24].

Figure 3b shows the capacitance of the cell membrane for the collagen-coated substrate. The results show a decrease from day 1 to day 3 for the control group. However, the capacitance starts to increase from day 4, when a monolayer forms. The change post-confluency suggests an increasing trend, with small variations between samples. On the other hand, the electrical capacitance of the forskolin-treated samples showed a slight increasing trend from day 1 to day 4, followed by a decrease. Electrical capacitance has been shown to be cell-dependent, which can cause conflicting results between studies [39]. Our study shows that the cell capacitance for the control wells increased throughout the days. However, it decreased for the forskolin-treated samples. We hypothesize that this phenomenon can be associated with a decrease in the cell surface area. When BeWo cells undergo differentiation and start to fuse, the nuclei area increases, as reported in a study by Blundell et al., in which the nuclear area of BeWo cells undergoing forskolin-induced differentiation was shown to increase from 1000 to 1500 pixels over a 48 h period [1]. This suggests a decrease in the cell membrane surface area.

### 3.3. FEA Simulation

#### 3.3.1. Validation of COMSOL Model

**Figure 4:**
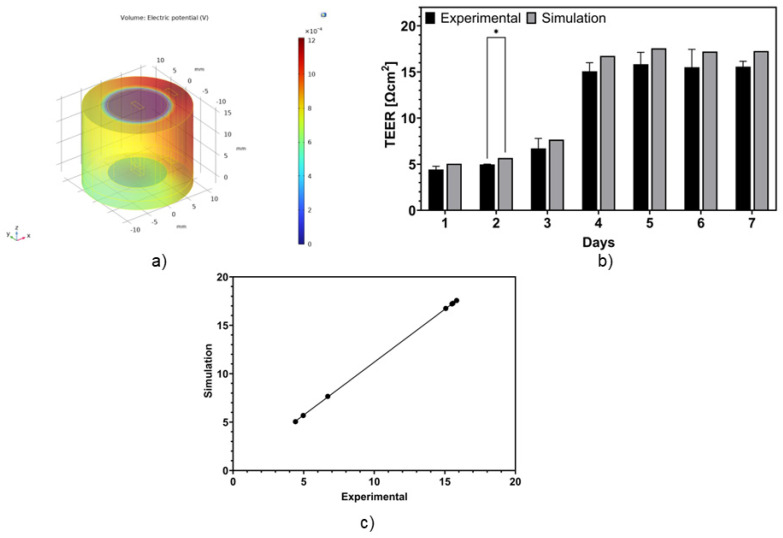
COMSOL multiphysics model results: (**a**) the potential distribution across the transwell setup; (**b**) the TEER values from the experiments, compared with the simulation results; and (**c**) the correlation plot between the simulation output and the experimental results. On the one hand, a comparison to the unfused cells will result in a decrease in capacitance. On the other hand, due to the multiple layer formation for the control group, the overall membrane surface area will increase. This phenomenon will result in an increase of the capacitance * indicates *p* ≤ 0.05.

By comparing the TEER values obtained from both the COMSOL model and the experimental model, shown in Figure 4a, we can validate the simulation model by plotting a correlation graph between the simulation results and the experimental results and by looking at the standard deviation of the experimental model. Figure 4b shows the correlation plot between the simulation and the experimental model, and we can conclude that both results align properly. In addition, looking at Figure 4c, the statistical analysis shows no significant differences between the simulation output and the experimental results, except for day 2. This can be explained by the fact that the simulation assumes a confluent monolayer regardless of the day, while the monolayer obtained experimentally is only present on day 4, as shown in the immunofluorescence analysis. While there are no significant differences between the other days, the simulation does not align perfectly with the experimental results. However, this can be explained by the fact that the media composition differs between the simulation and the experiment.

#### 3.3.2. Factors Affecting TEER Values

Given the validation of the COMSOL model, this model was then used to study the effect of various parameters on the TEER values, to explain the potential reasons for the wide range of TEER values reported across the literature. Liu et al. reported TEER values of 100–150 Wcm^2^, while Rothbauer et al. reported values of 0–65 Wcm^2^ [3,19]. The reason for this large variation has not been fully explained. However, using the COMSOL model, we intend to explore some of the potential reasons for such inconsistencies. The parameters tested on the model include the changing positions of the inner and outer electrodes, the depth of the electrodes, the porosity of the transwell insert, and the orientation of both the inner and outer electrodes.

**Figure 5:**
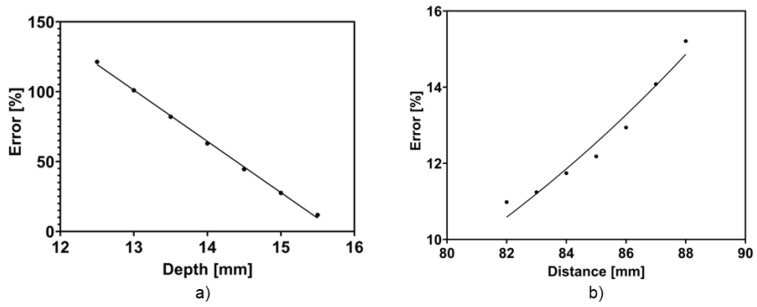
Error graph and fit of varying electrodes positions: (**a**) The depth of electrode in the basolateral chamber relative to the support. (**b**) The distance of the electrode in the apical chamber, relative to the second electrode.

First, the depth of the two electrodes was varied from 15.5 mm to 12.5 mm and the error was plotted as shown in Figure 5a. This resulted in a significant change in the TEER from 17.71 Wcm^2^ to 35.06 Wcm^2^, compared to the expected 15.84 Wcm^2^ from day 5, representing a substantial 97% difference in the obtained TEER value. This acts as one potential source of the variation in TEER measurements from one study to another.

Next, the percentage TEER error calculated by varying the distance between the two electrodes from 8.2 mm to 8.8 mm is shown in Figure 5b. The reference distance is 7.5 mm, as was used in the experimental model. A significant change was observed in the TEER, changing from 23.23 Wcm^2^ to 16.66 Wcm^2^ on day 5, when the experimental TEER value was 15.84 Wcm^2^, representing a significant variation of 39.4%.

Other parameters, such as the membrane’s porosity, outer electrode position, the angle of outer and inner electrodes, and the surrounding fluid’s conductivity were studied too. However, the error in TEER measurement introduced by these changes do not seem to be significant to explain the differences appearing in the literature. For example, changes in the basolateral electrode angle resulted in an 8% TEER change, whereas changes in the porosity of the membrane resulted in a 4% change in TEER values. Nevertheless, a combination of changes in these parameters could cause a noticeable change in TEER.

## 4. Conclusions

In conclusion, we showed that the AD2 device can be used to measure and track changes in placenta barrier permeability in vitro, namely the TEER and capacitive properties of the BeWo cell line. It was first concluded using immunofluorescence imaging that the cell fusion index of the BeWo cells undergoing terminal differentiation increased five times compared to those in an undifferentiated state, aligning to our current understanding of the syncytialization process. Second, the impedance spectroscopy measurement confirmed the formation of a confluent monolayer and the integrity of the cell layer, a method that allows for the assessment of this critical biological process without hindering the cell function. Finally, the electrical capacitance of the BeWo cells was measured. The cell capacitance is an electrical property that is dependent on the cell membrane surface area. The measurements show that upon the formation of a confluent monolayer, the electrical capacitance of the BeWo cells decreased when undergoing forskolin-induced differentiation/syncytialization, compared to those in an undifferentiated state. Therefore, we showed and validated that this cost-effective BIS system can be used to measure and track the placenta barrier permeability changes, namely the TEER and capacitive properties of the BeWo b30 cell line.

Finally, we validated the developed COMSOL model, based on the experimental approach. There was no statistical significance between the output of the COMSOL model and the experimental approach, allowing us to conclude that the model is suitable for future use, allowing us to deepen our understanding of influencing factors on TEER. Lastly, the developed COMSOL model shows that the large variation in the TEER results obtained in the literature is not just due to different cell growth conditions but also electrode geometrical variations, including the depth and orientation of the electrodes. A standardized support, similar to the one developed for our study, can be used to obtain more comparable results. Furthermore, the developed COMSOL model can be used to further study the current flow characteristics and optimize the transwell setup to minimize the scope of errors in TEER measurements, in addition to the already proven potential error sources.

## Figures and Tables

**Table 1:** The conductivity parameters used to build the COMSOL simulation model.

Material	Conductivity (S/m)
Semi-permeable membrane	0.154
PBS	1.54

## Data Availability

The datasets used and/or analyzed during the current study are available from the corresponding author on reasonable request.
